# Low-Temperature Growth of InGaAs Quantum Wells Using Migration-Enhanced Epitaxy

**DOI:** 10.3390/ma17040845

**Published:** 2024-02-09

**Authors:** Linsheng Liu, Ruolin Chen, Chongtao Kong, Zhen Deng, Guipeng Liu, Jianfeng Yan, Le Qin, Hao Du, Shuxiang Song, Xinhui Zhang, Wenxin Wang

**Affiliations:** 1 Guangxi Key Laboratory of Brain-Inspired Computing and Intelligent Chips/Key Laboratory of Integrated Circuits and Microsystems (Education Department of Guangxi Zhuang Autonomous Region), School of Electronic and Information Engineering/School of Integrated Circuits, Guangxi Normal University, Guilin 541004, China; linshengliu@mailbox.gxnu.edu.cn (L.L.);; 2 State Key Laboratory of Superlattices and Microstructures, Institute of Semiconductors, Chinese Academy of Sciences, Beijing 100083, China; 3 Institute of Physics, Chinese Academy of Sciences, Beijing 100190, China; 4 School of Physical Science and Technology, Lanzhou University, Lanzhou 730000, China; 5 Sino Nitride Semiconductor Co., Ltd., Dongguan 523000, China

**Keywords:** migration-enhanced epitaxy, low-temperature growth, InGaAs quantum wells

## Abstract

The growth of InGaAs quantum wells (QWs) epitaxially on InP substrates is of great interest due to their wide application in optoelectronic devices. However, conventional molecular beam epitaxy requires substrate temperatures between 400 and 500 °C, which can lead to disorder scattering, dopant diffusion, and interface roughening, adversely affecting device performance. Lower growth temperatures enable the fabrication of high-speed optoelectronic devices by increasing arsenic antisite defects and reducing carrier lifetimes. This work investigates the low-temperature epitaxial growth of InAs/GaAs short-period superlattices as an ordered replacement for InGaAs quantum wells, using migration-enhanced epitaxy (MEE) with low growth temperatures down to 200–250 °C. The InAs/GaAs multi-quantum wells with InAlAs barriers using MEE grown at 230 °C show good single crystals with sharp interfaces, without mismatch dislocations found. The Raman results reveal that the MEE mode enables the growth of (InAs)_4_(GaAs)_3_/InAlAs QWs with excellent periodicity, effectively reducing alloy scattering. The room temperature (RT) photoluminescence (PL) measurement shows the strong PL responses with narrow peaks, revealing the good quality of the MEE-grown QWs. The RT electron mobility of the sample grown in low-temperature MEE mode is as high as 2100 cm^2^/V∗s. In addition, the photoexcited band-edge carrier lifetime was about 3.3 ps at RT. The high-quality superlattices obtained confirm MEE’s effectiveness for enabling advanced III-V device structures at reduced temperatures. This promises improved performance for applications in areas such as high-speed transistors, terahertz imaging, and optical communications.

## 1. Introduction

InGaAs quantum wells (QWs) epitaxially grown on InP are an excellent optoelectronic material, which can be used to manufacture infrared photodetectors, lasers, high-speed transistors, and other optoelectronic devices widely used in optical communications, medical devices, and LiDAR [1,2,3,4,5]. In addition, with the development of THz technology, THz photoconductive antenna (PCA) prepared with low-temperature-grown InGaAs can be applied in the fields of security detection, material analysis, and medical imaging [6,7,8,9,10,11].

The critical parameters that mainly affect the growth and corresponding performance of InGaAs QWs are mainly indium composition, growth temperature, QW thickness, alloy scattering, and interface quality. Indium composition directly affects the QW bandgap, strain, and carrier confinement in QWs. In_0_._53_Ga_0_._47_As, which is lattice matched to an InP substrate and features a 0.74 eV bandgap, exhibits an excellent near-infrared optical response, leading to a wide range of applications in optoelectronics [5,12,13]. The growth temperature mainly affects the interface quality and material defects. Typical growth temperatures for InGaAs QWs in the conventional MBE growth mode are in the range of 400–500 °C [14]. The thickness of the QW determines the quantum confinement strength and electronic/optical properties, depending on the application requirements. The random arrangement of In and Ga in InGaAs leads to alloy scattering [15,16]. The sharpness of the interfaces between the QWs and the barrier layers are important for minimizing defect states and optimizing carrier mobility. This paper focuses on the low-temperature growth of lattice-matched InGaAs multiple QWs on InP substrates, mainly for high-speed device applications such as THz PCA. Therefore, the impact of low-temperature growth on the QW crystal quality, mobility, and lifetime will be mainly discussed.

The electron mobility of the InGaAs QWs is limited not only by polar optical phonon scattering and ionizing impurity scattering, but also by disorder scattering due to the random arrangement of atoms such as In and Ga. This disorder scattering greatly reduces the electron mobility [15,16].

In conventional molecular beam epitaxy (MBE) mode, the growth of InGaAs quantum wells normally requires the substrate temperature to be controlled in the range of 400–500 °C. In this temperature range, the diffusion of the dopant atoms as well as the roughening of the quantum well interfaces may severely affect the device performance. To address these issues, lowering the substrate temperature during growth is an effective strategy. However, it is difficult to achieve high-quality InGaAs quantum well growth at lower substrate temperatures using conventional MBE methods.

Leh Woon Lim et al. grew pin photodiodes with an InGaAs active layer at high (500 °C) and low (250 °C) temperatures. The experimental results showed that the photodiodes grown at 500 °C had a dark current density six orders of magnitude lower than those grown at 250 °C [14]. The increase in the dark current can be attributed to the shorter carrier lifetime, which is caused by the reduced growth temperature [14]. Additionally, the photodiodes grown at low temperatures exhibit lower photoresponsivity. This is caused by the reduced diffusion length of the minority carriers, which is related to the poorer crystal quality of the layer grown at low temperatures. High-temperature annealing provides a pathway to restore the photoresponsivity [14]. Compared to the high temperature growth of InGaAs, the low-temperature grown InGaAs exhibits a reduced carrier lifetime, making it more suitable for applications in high-speed devices, such as femtosecond optically excited THz PCAs [17].

The increase in arsenic antisite defects during growth at lower temperatures is primarily due to the reduced atomic mobility at these temperatures, which causes arsenic atoms to more readily occupy non-ideal sites, such as the gallium site, thus forming arsenic antisite defects. These defects act as non-radiative recombination centers capable of trapping the carriers and consequently shortening the effective lifetime of the carriers [12].

InAs/GaAs short-period superlattices (SPSs) are able to be grown on InP substrates owing to the approximate lattice-match in between the two. In InGaAs alloys, the In and Ga atoms of group III are random. This randomness induces additional electron scattering, which reduces the electron mobility. Conversely, InAs/GaAs SPSs consist of alternating one or several InAs and GaAs single-atom layers. The ordered arrangement of In and Ga atoms in these superlattices suppresses alloy scattering due to the random occupancy of lattice sites by In and Ga atoms in InGaAs. Through avoiding this randomness, alloy scattering is mitigated by using InAs/GaAs superlattices instead of InGaAs alloys [16,18]. These SPSs, as ordered replacements for InGaAs random alloys, may help to reduce the effects of alloy scattering in InGaAs-based high-speed electronic devices [15,16,19]. Andre et al. used metal–organic vapor phase epitaxy (MOVPE) to grow (InAs)n(GaAs)n short-period superlattices, attaining mobilities slightly above those of InGaAs [19]. For this reason, InAs and GaAs SPSs will be used in this paper instead of the InGaAs alloy.

(InAs)_n_(GaAs)_n_ short-period superlattices grown at common substrate temperatures (400–500 °C) using the conventional MBE growth mode show three-dimensional growth when n is greater than or equal to three, due to the limitation of the critical thickness. Many mismatch dislocations appeared in 100–170 pairs of (InAs)_2_(GaAs)_2_ superlattices grown at substrate temperature of 500 °C, as reported by Cheng et al. [20].

To achieve high-quality InAs/GaAs short-period superlattices on InP substrates, the use of the migration-enhanced epitaxy (MEE) growth mode and a lower growth temperature is an effective strategy. MEE technology offers the possibility to grow high-quality InGaAs quantum wells at substrate temperatures as low as 200 °C, and the grown materials exhibit excellent optical properties. During the MEE process, (In) Ga, and As are supplied by alternately turning on and off the corresponding shutters. During the shutter closure of As, the pressure of As is significantly reduced by at least one order of magnitude, and this pressure reduction significantly increases the diffusion length of Ga atoms at the surface, even at low substrate temperatures. In this way, the growth of high-quality quantum well structures can be achieved while controlling the diffusion of dopant atoms and maintaining the interface flatness of the quantum well, which is important for the fabrication of high-performance optoelectronic devices. Due to these advantages, the MEE mode has been widely used in the growth of III-V materials [21,22,23,24].

The crystal quality of InAs/GaAs short-period superlattices was effectively improved by lowering the growth temperature and adjusting the atomic layer numbers of InAs and GaAs. Gerard et al. utilized MEE’s technique to grow a 130 nm (InAs)_4_(GaAs)_3_ short-period superlattice on an InP (100) substrate at a substrate temperature of 370 degrees. Scanning transmission microscopy of the cross section of the grown sample revealed a well-defined superlattice period, and no mismatch dislocations were observed [25].

In the study of temperature control for the low-temperature growth on InP substrates, so far, the conventional mode growth of InGaAs has received more research attention [12,26,27,28,29,30,31,32], while the growth of InAs/GaAs short-period superlattices in the MEE mode has been relatively less investigated [15,25,33].

For the low-temperature growth of InGaAs quantum wells using conventional methods, Deyan Dai et al. grew InGaAs/InAlAs multiple quantum wells in the temperature range of 230–280 °C. Due to the low growth temperatures, X-ray diffraction (XRD) measurements show only one satellite peak, suggesting some degradation in the crystal quality [12]. G. B. Galiev and colleagues grew 100 cycles of InGaAs/InAlAs quantum wells at 200 °C. Transmission electron microscopy (TEM) characterization revealed a significant density of defects including twins, stacking faults, and small-angle domains [26]. The migration length of adatoms decreases significantly at temperatures around 200 °C [34]. As a result, the quality of the crystals grown below 200 °C deteriorates. Therefore, the study of low-temperature growth primarily focuses on temperatures above 200 °C [12].

To the best of our knowledge, up to now, studies on the growth of InAs/GaAs short-cycle superlattices using the MEE growth mode on an InP(100) substrate have mainly focused on the growth temperature range of 350–400 °C [15,25,33]. There are very limited studies conducted for growth temperatures below 350 °C and, in particular, there are no studies available for growth temperatures in the 200–250 °C range. Growth at such low temperatures results in an increase in arsenic antisite defects (As_Ga_ defects) and in the arsenic content in the material. This increase contributes to shorter carrier lifetimes and facilitates the fabrication of high-speed devices such as terahertz PCAs and high-speed optical switches.

In addition, in practical applications it is often necessary to achieve better light absorption by the material, and thicker InAs/GaAs superlattice layers need to be grown, which is also a challenge. In this paper, we use InAs/GaAs short-period superlattices instead of InGaAs as quantum potential wells and InAlAs as barriers to grow multi-quantum well structures at a substrate temperature of 230 °C (thermocouple temperature). Via comprehensive HRTEM, Raman, PL, Hall, and carrier lifetime measurements, our results demonstrate the successful growth of InAs/GaAs short-period superlattices under low-temperature conditions. This will provide a valuable reference and new insight for material design and optoelectronic device applications.

## 2. Materials and Methods

The materials utilized in this study were fabricated using the FW-VI-60 model of the MBE system manufactured by SKY Technology Development Co., Ltd., Shenyang, China. Sample A was grown on a semi-insulating InP (100) substrate with a 12 nm thick (InAs)_4_(GaAs)_3_ quantum well layer using the MEE growth mode. The MEE growth mode is the periodic alternating switching of Ga (or In) and As shutters during the growth of GaAs or InAs layers. A barrier layer of In_0_._52_Al_0_._48_As, also 12 nm in thickness, was formed, to achieve lattice matching with the InP substrate. The structure included 50 repetitions of the quantum well, and the epitaxial growth was conducted at a thermocouple-measured temperature of 230 °C. Both the well and the barrier regions were doped with beryllium (Be) to a concentration of 2 × 10^18^ cm^−3^ to increase material resistivity. Prior to the samples’ growth, Fe-doped, semi-insulating InP (100) substrates were deoxidized at 520 °C (thermocouple temperature) under As protection.

For comparison, control Sample B was grown on a semi-insulating InP (100) substrate through the conventional MBE growth mode. The quantum well layer is 12 nm thick and consists of In_0_._53_Ga_0_._47_As. The barrier layer of sample B consists of In_0_._52_Al_0_._48_As with a thickness of 12 nm and is lattice matched to the InP substrate. The quantum well also comprises 50 periods, with the growth temperature maintained at 230 °C, as measured by a thermocouple. Doping with Be was applied to both the well and the barrier with a concentration of 2 × 10^18^ cm^−3^.

The structural analysis of the prepared superlattice was conducted using the high-angle annular dark-field scanning transmission electron microscopy (HAADF-STEM) technique, specifically with the Thermofisher Talos F200X instrument (Waltham, MA, USA). The HAADF-STEM method was used to precisely characterize the superlattice. The samples required for the cross-sectional observation by HAADF-STEM were prepared using the focused ion beam (FIB) technique.

Electrical parameters such as sample mobility and resistivity were characterized using Hall effect measurements using the HL5500 system from Nanometrics Hall Measurement. In addition, the carrier lifetime at room temperature was determined using the pump–probe transient reflectivity (Δ*R*/*R*) method. For this purpose, an optical parametric amplifier (OPA, Opera Solo, Coherent Inc., Sunnyvale, CA, USA) pumped by a Ti: Sapphire regenerate amplifier (Legend Elite, Coherent Inc., Sunnyvale, CA, USA) was used. The output from the OPA had a pulse width of ~150 fs and a repetition frequency of 1 KHz. The polarization of both the pump and probe beams was linear, and the wavelength was tuned to approach the bandgap of superlattice near 1.45 µm.

The room temperature (RT) Micro-Raman spectra of the samples were measured using Horiba’s LabRAM HR Evolution under an excitation of 532 nm with a power of 2.280 mW (measured using a Thorlabs S130C Optical Power Meter, Newton, NJ, USA). The RT micro-photoluminescence (Micro-PL, OmniFluo-FLIM from Zolix Instruments Co., Ltd., Beijing, China) spectroscopy of the samples was conducted with an excitation wavelength of 980 nm and a power of 27 mW, via using a MicroPL spectrometer (the OmniFluo-FLIM from Zolix Instruments Co., Ltd., Beijing, China), which is equipped with a near-infrared photomultiplier tube (Hamamatsu, FLS-NIRPMT01-MS, Shizuoka Pref., Japan). The crystalline structure and quality of the superlattice were characterized using a high-resolution X-ray diffractometer (HRXRD, PANalytical, Malvern, UK, X’Pert3 MRD XL, Cu Kα1, λ = 1.54056 Å) in this study.

## 3. Results and Discussion

### 3.1. TEM of Low-Temperature-Grown (InAs)_4_(GaAs)_3_/InAlAs Multiple Quantum Wells Using the MEE Growth Mode

In this study, a 50-cycle multiple quantum well (MQW) sample, denoted as sample A, was epitaxially grown on an Fe-doped, semi-insulating InP (100) substrate at a low temperature of 230 °C (thermocouple temperature). The MQW structure in sample A consisted of a 12 nm thick (InAs)_4_(GaAs)_3_ quantum well layer, which was grown using the MEE growth mode. In addition, a 12 nm thick In_0.52_Al_0.48_As layer was employed in sample A as a barrier layer for the quantum well.

**Figure 1:**
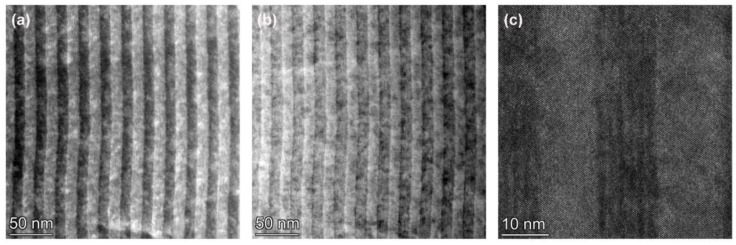
(**a**) HAADF-STEM images of low-temperature-grown 12 nm (InAs)_4_(GaAs)_3_/12 nm InAlAs multiple quantum wells sample A; (**b**) Bright-field scanning transmission electron microscopy (BF-STEM) image of sample A; (**c**) High-resolution TEM of sample A.

Figure 1a–c show the HAADF-STEM, bright field, and high-resolution TEM images of the low-temperature-grown 12 nm (InAs)_4_(GaAs)_3_/12 nm InAlAs MQWs in sample A, respectively. As seen in Figure 1, the quantum wells exhibit a well width and a barrier width of 12 nm, a pronounced period, and a steep interface. The TEM results presented in Figure 1 indicate a good crystalline quality of the MQW sample, with no discernible defects detected. Notably, the high-resolution TEM image in Figure 1c reveals a distinct periodicity in the (InAs)_4_(GaAs)_3_ short-period superlattice region, as opposed to a randomized InGaAs alloy. This structured region comprises alternating sequences of four monolayers (MLs) of InAs and three MLs of GaAs, respectively. The interfaces between these materials are abrupt and do not exhibit significant mismatch dislocations. This is expected to enhance the optoelectronic properties of the material.

**Figure 2:**
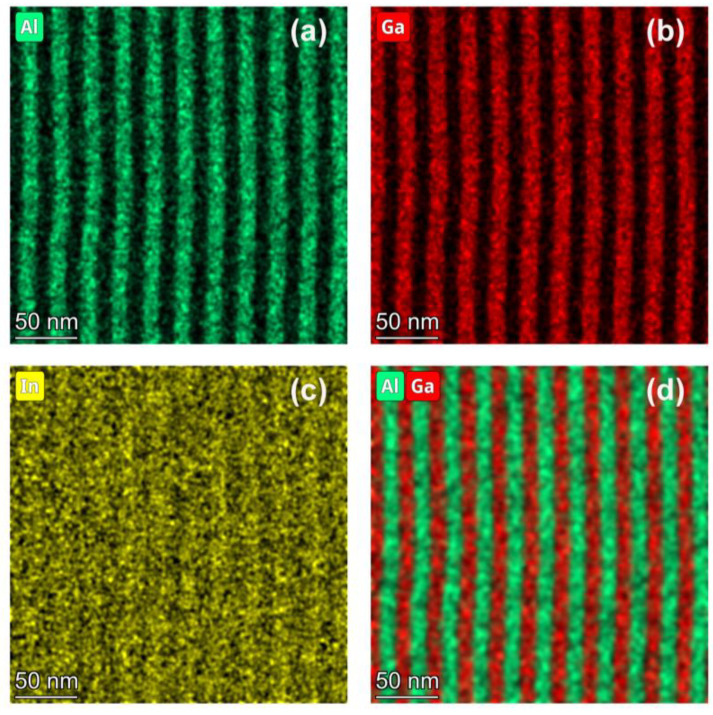
STEM-energy dispersive X-ray spectroscopy (STEM-EDS) derived elemental distribution maps for low-temperature-grown 12 nm (InAs)_4_(GaAs)_3_/12 nm InAlAs multiple quantum wells, sample A. (**a**) Al, (**b**) Ga, (**c**) In, and (**d**) the overlay of Al and Ga distributions.

Figure 2 shows the STEM-EDS results of low-temperature-grown 12 nm (InAs)_4_(GaAs)_3_/12 nm InAlAs MQWs (sample A). The EDS maps reveal a pronounced periodicity in the distribution of Al and Ga across the quantum wells, with sharp interfaces and no discernible elemental segregation. As seen in Figure 2a,b, the Al and Ga concentration profiles exhibit clear periodic variations corresponding to the quantum well growth sequence, with steep distribution boundaries. Although the periodicity of In is less distinct in the image shown in Figure 2c, it is still discernible. This periodicity arises due to the alternate layer growth of InAs and GaAs in the (InAs)_4_(GaAs)_3_ structure grown using the MEE mode. Owing to its lower spatial resolution compared to the high-resolution TEM imaging in Figure 1c, the STEM-EDS fails to resolve the In distribution within the quantum well with atomic layer precision. Therefore, the In distribution within the quantum wells, as depicted in Figure 2c, does not show the multilayered structure phenomenon visible in the high-resolution TEM image in Figure 1c. The distribution of Ga elements in the quantum well in Figure 2b does not show the multilayered structure as in Figure 1c for the same reason. Figure 2d presents an overlay of the Al and Ga elemental distributions. It shows a perfect spatial alternation of Al in the InAlAs quantum barriers and Ga in the (InAs)_4_(GaAs)_3_ quantum wells. The interfaces are observed to be abrupt, with no atomic segregation evident.

### 3.2. TEM of Low-Temperature-Grown InGaAs/InAlAs Multiple Quantum Wells Using the Conventional MBE Growth Mode

**Figure 3:**
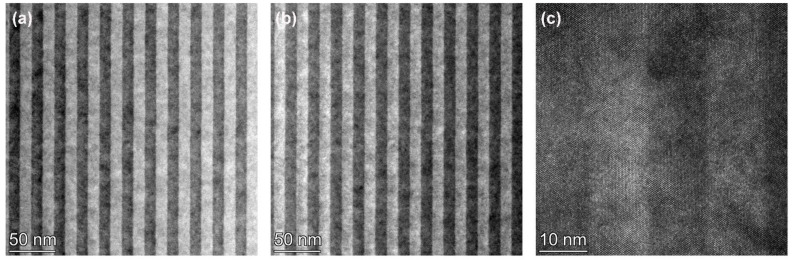
(**a**) HAADF-STEM images of low-temperature-grown 12 nm InGaAs/12 nm InAlAs multiple quantum wells sample B; (**b**) BF-STEM image of sample B; (**c**) High-resolution TEM of sample B.

Figure 3a–c present HAADF-STEM, BF-TEM, and high-resolution TEM images of sample B, comprising 12 nm InGaAs/12 nm InAlAs multi-quantum wells grown at a substrate temperature of 230 °C (thermocouple temperature) using the conventional MBE mode. Figure 3 shows that the quantum wells of sample B have a pronounced period, a steep interface, good crystallization quality, and no significant defects are found. No multilayer structure similar to that shown in Figure 1 is observed in the InGaAs quantum well in Figure 3a–c, which is due to the fact that sample B is grown in a conventional MBE growth mode and the InGaAs is not grown in an atomic layer-by-atomic layer manner, similar to that of MEE.

**Figure 4:**
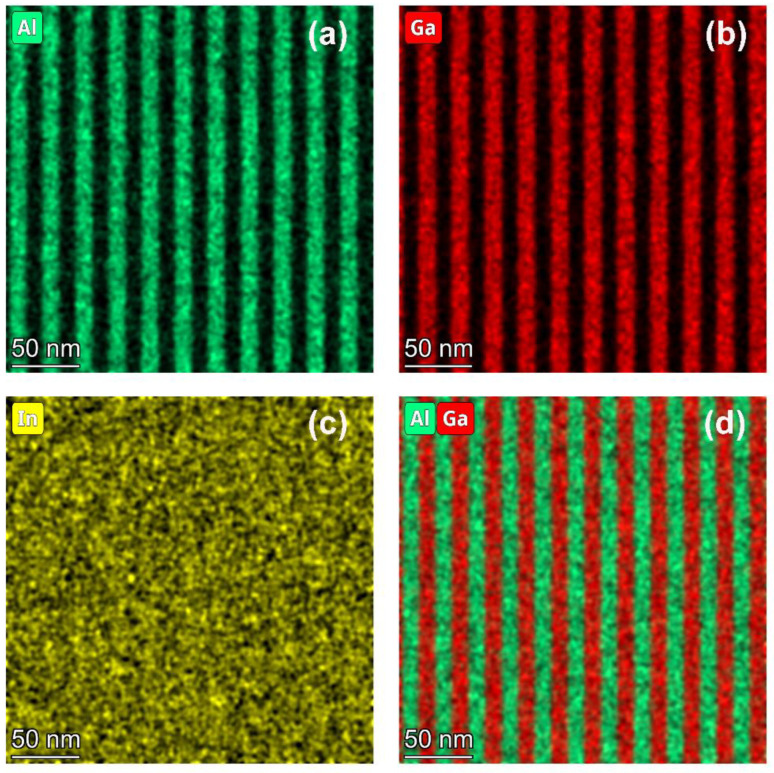
STEM-EDS derived elemental distribution maps for low-temperature-grown 12 nm InGaAs/12 nm InAlAs multiple quantum wells, sample B. (**a**) Al, (**b**) Ga, (**c**) In, and (**d**) the overlay of Al and Ga distributions.

Figure 4a,b show the STEM-EDS results for a low-temperature-grown 12 nm InGaAs/12 nm InAlAs multiple quantum well. The EDS results show a clear periodicity in the distribution of Al and Ga elements in the quantum well, with a steep interface, and no significant elemental segregation is found. Figure 4c presents a homogenous In distribution across the quantum wells, which contrasts with the periodicity observed for Al and Ga. In contrast to Figure 2c, sample B’s quantum wells are composed of InGaAs and InAlAs, with both the well and barrier regions incorporating indium. The In content varies by approximately 1% between the wells and barriers. However, due to the resolution limitations of EDS, this subtle difference is indistinguishable, resulting in an ostensibly uniform indium distribution as observed in Figure 4c. Additionally, the overlaid Al and Ga mapping in Figure 4d clearly shows the spatial separation between the InAlAs and InGaAs layers, with sharp interfaces and negligible atomic segregation.

### 3.3. Raman Measurements of Low-Temperature-Grown (InAs)_4_(GaAs)_3_/InAlAs and InGaAs/InAlAs MQWs

**Figure 5:**
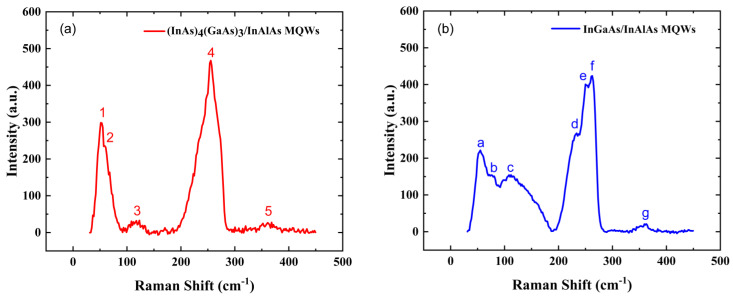
Raman spectra of low-temperature-grown multiple quantum wells: (**a**) 12 nm (InAs)_4_(GaAs)_3_/12 nm InAlAs MQWs, sample A; and (**b**) 12 nm InGaAs/12 nm InAlAs MQWs, sample B.

Raman spectroscopy is a powerful technique that can be used to characterize periodic structures such as semiconductor quantum wells or superlattices. Raman spectroscopy has been extensively utilized to elucidate the phonon properties intrinsic to superlattices and evaluate the interface quality in superlattices [35]. Figure 5a,b show the room temperature Raman spectra of 12 nm (InAs)_4_(GaAs)_3_/12 nm InAlAs MQWs (Sample A) and 12 nm InGaAs/12 nm InAlAs MQWs (Sample B), respectively. Longitudinal acoustic (LA) phonon modes, located at the center of the Brillouin zone, offer insights into the periodicity of the superlattice. LA phonon mode vibrational bands reflect the low-frequency vibrational modes between atoms or molecules within a material. The vibrational bands associated with the LA mode are typically found in the low wavenumber region, around 10–100 cm^−^^1^. LA modes can reveal low-frequency acoustic vibrations between different atomic layers within a superlattice or quantum well. These vibrations involve the longitudinal (along the growth direction of the multiple quantum wells) stretching or compressing of the entire lattice. For superlattices or multiple quantum wells, the vibrational properties of LA modes can also indicate the regularity and periodic changes in the arrangement of the atoms at the interface, thus providing crucial insights to study the superlattice or quantum well.

In the Raman spectrum of the low-temperature-grown 12 nm (InAs)_4_(GaAs)_3_/12 nm InAlAs multiple quantum wells, the peak observed at 52 cm^−1^ (labeled as “1”), is ascribed to the transverse acoustic (TA) phonon mode of InAs. Similarly, the peak marked as ‘a’ in the Raman spectrum of sample B is also attributed to the TA mode of InAs. The TA mode in InAs crystals refers to the vibrational mode where In and As atoms vibrate perpendicularly to the direction of the lattice vibration propagation.

The Raman peak labeled “2” belongs to the LA mode, which is related to the periodicity of the superlattice. The position of this peak is close to the LA mode position of the (InAs)_4_(GaAs)_3_ superlattice reported in the literature [18]. The position of the LA Raman peak can also be determined using the theoretical estimates provided in reference [36]. This peak originates from the (InAs)_4_(GaAs)_3_ quantum well grown in MEE mode. This peak was not observed in sample B.

The Raman peaks labeled as “3”, “b”, and “c“ in samples A and B are ascribed to the disorder-activated longitudinal acoustic (DALA) modes, consistent with the reports in the literature [37,38]. The molecular vibrational bands corresponding to the DALA modes reflect the vibrations of the atoms along the propagation direction but are affected by the structural disorder of the material. The presence and intensity of DALA peaks in Raman spectra are directly related to the degree of disorder inside the material. These bands are usually observed in the low-frequency region and are similar to the conventional LA modes, but are activated by the presence of disorder, thus distinguishing them from the conventional LA modes. The significantly weaker DALA peaks in sample A compared to sample B imply that the (InAs)_4_(GaAs)_3_/InAlAs MQWs grown using MEE have better atomic ordering than the InGaAs/InAlAs MQWs using conventional MBE. This suggests that MEE is a more efficient mode for achieving a well-ordered MQW structure.

The longitudinal optical (LO) phonon modes correspond to the lattice vibrations of the atoms moving parallel to the direction of phonon propagation. Stresses in the quantum well can have a significant effect on the LO phonon vibrational bands. The Raman peak observed at 255 cm^−1^ in sample A (labeled as “4”), is ascribed to the overlapping LO phonon modes of InAs and GaAs within the MEE-grown strained-layer superlattice structure of (InAs)_4_(GaAs)_3_. The compressive strain within the InAs superlattice results in a Raman shift towards higher wavenumbers, whereas the tensile strain in GaAs leads to a shift towards lower wavenumbers. The strain-induced opposing shifts ultimately result in the convergence and merging of the InAs and GaAs LO peaks into the observed 255 cm^−1^ Raman peak [39,40]. In sample B, the Raman peak at 233 cm^−1^ (labeled as “d”), is attributed to the LO mode of InAs, while the peak at 262 cm^−1^ (labeled as “f”), is attributed to the LO mode of GaAs, consistent with reports in the literature [40]. The peak between 240 and 250 cm^−1^, labeled “e”, is consistent with disordered activated optical phonons, also reported in previous studies [40,41]. The appearance of the peak labeled “e” in sample B indicates a considerable degree of atomic disorder within the InGaAs/InAlAs MQWs grown at low temperatures using the conventional MBE mode. This atomic-level disorder can induce significant alloy scattering that may negatively affect the carrier mobility in these low-temperature MQWs.

The AlAs-like modes here are LO phonon modes, corresponding to the relative vibrations between the Al and As atoms, which vibrate in the direction of phonon propagation. In samples A and B, the Raman peaks located at about 360 cm^−1^ (labeled as “5” and “g”) are attributed to AlAs-like modes, in agreement with those reported in the literature [38,42]. The Raman results for samples A and B reveal that the (InAs)_4_(GaAs)_3_/InAlAs MQWs grown using the MEE mode exhibit excellent periodicity. This well-defined periodic structure is effective in significantly reducing alloy scattering within the MQWs.

These findings were also confirmed via TEM results shown in Figure 1 and Figure 2, the high-quality periodic structure obtained using the MEE growth mode. Low-temperature-grown InGaAs/InAlAs MQWs exhibit abrupt interfaces based on TEM observations, with negligible evidence of misfit dislocations. Despite this structural sharpness at the interface, Raman spectroscopy reveals a pronounced disorder in the arrangement of group III atoms within the MQWs. This atomic disorder manifests as distinct peaks in the Raman spectra, indicative of considerable alloy scattering within the MQWs. As an excellent complement to TEM, Raman spectroscopy can be used as an effective means to check the crystal quality of low-temperature-grown MQWs.

### 3.4. MicroPL Spectra of Low-Temperature-Grown (InAs)_4_(GaAs)_3_/InAlAs and InGaAs/InAlAs MQWs

**Figure 6:**
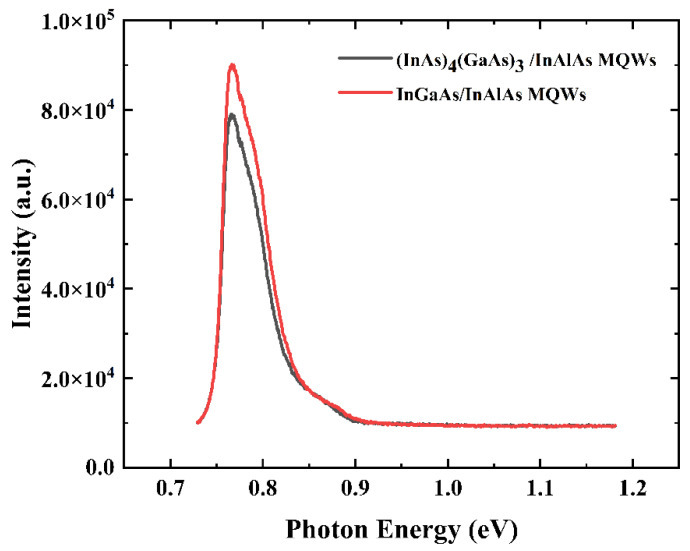
MicroPL spectra of low-temperature-grown (InAs)_4_(GaAs)_3_/InAlAs and InGaAs/InAlAs MQWs at RT.

MicroPL is able to measure the PL signal in a small region of a sample, and in combination with a high-quality near-infrared photomultiplier tube as a detector, it enables the efficient acquisition of the PL signal from low-temperature-grown materials. Figure 6 shows the RT microPL spectra of low-temperature-grown (InAs)_4_(GaAs)_3_/InAlAs and InGaAs/InAlAs MQWs. The peaks for both (InAs)_4_(GaAs)_3_/InAlAs and InGaAs/InAlAs MQWs are in the same position, corresponding to the band-edge emission of In_0_._53_Ga_0_._47_As. The PL peak position of the MQWs is at 0.765 eV, corresponding to the electronic transition between the first electron and heavy-hole levels (E1-HH1) in the quantum wells. The PL peak position of the MQWs in this study is at 0.765 eV, which is slightly smaller than the theoretical (0.7876 eV) and experimental (0.775 eV) values reported for room temperature In_0_._53_Ga_0_._47_As/In_0_._52_Al_0_._48_As MQWs in the literature [43]. This is due to the fact that 2 × 10^18^ cm^−^^3^ Be is doped into the MQWs in this work. According to [44], doping with Be (2 × 10^18^ cm^−^^3^) can narrow the bandgap of InGaAs by about 30 meV. Therefore, the PL peak positions of the MQWs in this work are reasonable considering the bandgap narrowing effect induced by Be doping. Gerard et al. studied the PL and PL excitation spectra of MQWs containing (InAs)_2_(GaAs)_2_, and the results showed that (InAs)_2_(GaAs)_2_ exhibits pseudo-alloy-like behavior [15]. Their further study revealed that the actual bandgap of the (InAs)_4_(GaAs)_3_ short-period superlattice is about 0.76 eV [45], close to the bandgap of In_0_._53_Ga_0_._47_As at room temperature (0.743 eV).

The PL intensities of the two MQWs are comparable, with full widths at half maximum (FWHM) of 53.5 meV and 54.5 meV, respectively. These results indicate that the (InAs)_4_(GaAs)_3_/InAlAs MQWs grown using the MEE mode at low temperatures exhibit a superior crystal quality and more abrupt interfaces compared to the InGaAs/InAlAs MQWs grown at low temperatures. The FWHM reflects the crystalline quality and structural properties of the quantum well material. In finite-sized quantum wells such as (InAs)_4_(GaAs_)3_/InAlAs and InGaAs/InAlAs MQWs, a non-abrupt interface in the quantum well and inhomogeneities in the thickness of the quantum wells can broaden the FWHM. In addition, the random distribution of In and Ga atoms within the InGaAs alloy contributes to the FWHM broadening. Additionally, the PL spectra exhibit a broad peak attributed to the high Be doping concentrations (2 × 10^18^ cm^−^^3^), resulting in a reduction in the semiconductor bandgap. This leads to the generation of a broad PL peak near the bandgap energy [44,46].

### 3.5. Hall Effect and Carrier Lifetime Measurements of Low-Temperature-Grown (InAs)_4_(GaAs)_3_/InAlAs and InGaAs/InAlAs MQWs

The mobility and the carrier lifetime are important parameters for the fabrication of high-speed optoelectronic devices such as THz PCAs. These parameters depend on the crystallization quality of the epitaxial material and the device structure. Hall effect measurements were performed on samples at room temperature to determine their electrical properties. The MQWs grown using MEE exhibited a mobility of 2100 cm²/Vs for the (InAs)_4_(GaAs)_3_/InAlAs structure. This is in contrast to the InGaAs/InAlAs MQWs, which were grown using a conventional MBE growth mode and demonstrated a mobility of 1840 cm²/Vs. For comparison, room temperature mobility for Be-doped low-temperature-grown InGaAs has been previously reported to be 425 cm²/Vs [8]. The observed increase in mobility within this study can be ascribed to the substitution of InGaAs with an (InAs)_4_(GaAs)_3_ short-period superlattice, which effectively diminishes lattice scattering by reducing the disorder typically present in ternary alloys.

**Figure 7:**
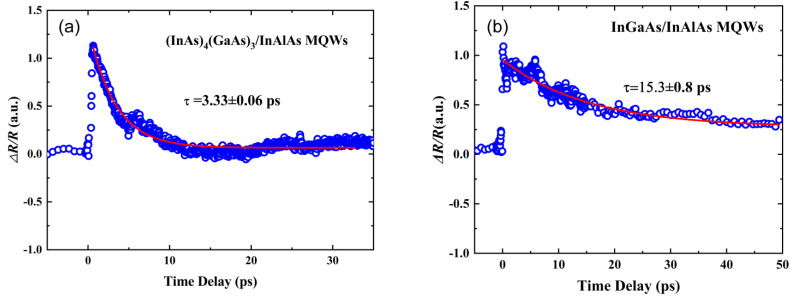
The room temperature pump–probe transient reflectivity results for low-temperature-grown multiple quantum wells with an excitation at 1450 nm, where the scattered blue circles represent the experimental data and the red solid line indicates the fit using a single exponential decay function: (**a**) 12 nm (InAs)_4_(GaAs)_3_/12 nm InAlAs MQWs; (**b**) 12 nm InGaAs/12 nm InAlAs MQWs.

Figure 7a,b show the room temperature pump–probe transient reflectivity results of low-temperature-grown 12 nm (InAs)_4_(GaAs)_3_/12 nm InAlAs and 12 nm InGaAs/12 nm InAlAs MQWs, respectively. Pump–probe transient reflectivity measurements were performed with an excitation wavelength of 1450 nm and a pump power of 6 mW. The decay traces provide a measure of the carrier lifetimes (τ) within the MQWs and reveal the recombination behavior of the carriers in the samples. The carrier lifetime (τ) of the MQW is indicated by the red decay curve fitted using a single exponential decay function. The carrier lifetimes of (InAs)_4_(GaAs)_3_/InAlAs and InGaAs/InAlAs MQWs were determined to be 3.3 ps and 15.3 ps, respectively. The carrier lifetime of a Be-doped InGaAs sample grown at low temperature on a semi-insulating InP substrate was measured to be 7.8 ps at 250 °C by Namje Kim et al. [47]. The data indicate that the (InAs)_4_(GaAs)_3_/InAlAs MQWs grown in this study possesses a markedly reduced carrier lifetime. The reduced carrier lifetime of the (InAs)_4_(GaAs)_3_/InAlAs MQWs grown using a low-temperature MEE mode can be attributed to the atomic layer-by-layer growth. Specifically, during quantum well deposition, slower growth rates and longer arsenic shutter openings lead to an increase in arsenic entry into MQWs at low temperatures. This leads to a higher density of antisite defects, i.e., arsenic on gallium sites (As_Ga_), acting as non-radiative recombination centers that decrease the carrier lifetime.

In Figure 6, the PL intensity of the (InAs)_4_(GaAs)_3_/InAlAs MQWs at RT is slightly lower than that of the InGaAs/InAlAs MQWs, which may be due to an increase in non-radiative recombination centers. The longer carrier lifetime observed in InGaAs/InAlAs MQWs can be attributed to a lower incorporation of excess arsenic during growth, resulting in a lower concentration of non-radiative recombination centers.

### 3.6. HRXRD of Low-Temperature-Grown (InAs)_4_(GaAs)_3_/InAlAs and InGaAs/InAlAs MQWs

**Figure 8:**
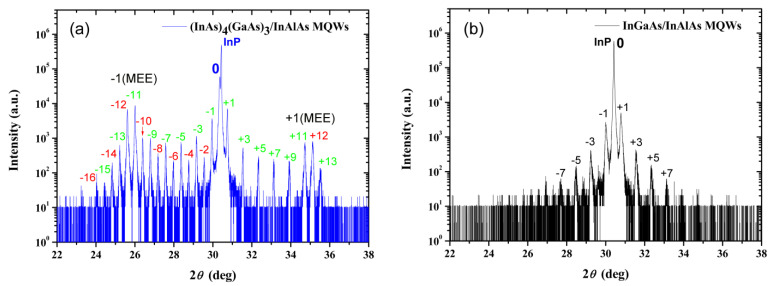
HRXRD patterns of low-temperature-grown multiple quantum wells obtained in the *θ*/2*θ* scanning mode: (**a**) (InAs)_4_(GaAs)_3_/InAlAs MQWs; and (**b**) InGaAs/InAlAs MQWs.

As shown in Figure 8a,b, for both samples of low-temperature-grown (InAs)_4_(GaAs)_3_/InAlAs and InGaAs/InAlAs MQWs, the 2θ peak position of the InP substrate is at 30.43°, which matches the theoretical diffraction peak position of the InP (002) crystal plane [19,48]. Shown in Figure 8a, the HRXRD results of (InAs)_4_(GaAs)_3_/InAlAs MQWs grown at low temperatures in an MEE mode show several key features. First, the 0th-order satellite peak is quite close to the InP substrate peak, indicating that the epitaxial layer of MQWs is lattice matched to the InP substrate. Lattice matching is crucial for achieving high-quality epitaxial layers, as it reduces defects and improves device performance. It is worth noting that the satellite peak pattern consists of two different sets of satellite peaks. One set is formed by the 12 nm (InAs)_4_(GaAs)_3_/12 nm InAlAs MQWs, which are labeled with numbers in Figure 8a. The other set of satellite peaks is formed by the (InAs)_4_(GaAs)_3_ layer grown using an MEE mode at low temperature, labeled as −1(MEE) and +1 (MEE). The experimental results show that the satellite peaks formed by the 12 nm (InAs)_4_(GaAs)_3_/12 nm InAlAs MQWs are highly visible and widely distributed (from −16th to +13th order). In addition, the narrow FWHMs of the satellite peaks reflect the high degree of periodicity in the MQWs.

By analyzing the satellite peak intervals and applying the theory from reference [49], the periodicity of the quantum wells is calculated to be 24.10 nm, which is consistent with the design value. This calculation takes into account the total thickness of both the (InAs)_4_(GaAs)_3_ and InAlAs layers. Similarly, by analyzing the +1 (MEE) and −1 (MEE) satellite peaks in the (InAs)_4_(GaAs)_3_ layer grown using a low-temperature MEE mode, the single-period thickness of (InAs)_4_(GaAs)_3_ is calculated to be 2.07 nm. This is in agreement with the result of 2.06 nm calculated from the XRD satellite peaks reported in the literature [25]. In the structure of (InAs)_4_(GaAs)_3_/InAlAs MQWs, the (InAs)_4_(GaAs)_3_ layer serving as the quantum well consists of six such periods, thus having a total thickness of 12.42 nm, which is consistent with the design value. The (InAs)_4_(GaAs)_3_/InAlAs MQWs show multiple satellite peaks, reflecting the periodic arrangement in the MQWs, and the quantum well interface is steep. Satellite peaks of (InAs)_m_(GaAs)_n_ short-period superlattices were also observed in previous reports [19,48].

Furthermore, the satellite peaks of the (InAs)_4_(GaAs)_3_/InAlAs MQWs are asymmetric on both sides of the InP substrate, and most of the even-order satellite peaks on the right side of the InP substrate are nearly indistinguishable. The extinction of the even-order peaks to the right of the InP substrate peaks is related to the fact that the thicknesses of the (InAs)_4_(GaAs)_3_ and InAlAs layers in the (InAs)_4_(GaAs)_3_/InAlAs MQWs are essentially the same. Figure 8b shows that the equal thicknesses of the InGaAs and InAlAs layers lead to most even-order peaks being invisible. The even-order satellite peaks of (InAs)_4_(GaAs)_3_/InAlAs MQWs on the left side of the InP substrate exist but are weaker in intensity than the odd-order peaks. This could be attributed to changes in the lattice symmetry of the quantum wells, strain within the quantum wells, and the thicknesses of (InAs)_4_(GaAs)_3_ and InAlAs layers not being exactly the same. Due to the use of (InAs)_4_(GaAs)_3_ grown in an MEE mode instead of InGaAs as the well material changes the lattice symmetry [15], which might be the reason why even-order satellite peaks do not extinguish. The HRXRD results show that the 0th-order satellite peak, although close to the InP substrate peak, does not overlap, indicating the possible presence of strain in the quantum wells. Additionally, the Raman results in Figure 5a also confirm the presence of strain in the (InAs)_4_(GaAs)_3_/InAlAs MQWs, which may impact the extinction of even-order peaks. Theoretical calculations show that the thicknesses of the (InAs)_4_(GaAs)_3_ and InAlAs layers are close, but they are not identical. This may also lead to the appearance of even-order satellite peaks.

Comparing Figure 8a,b, it can be seen that under low-temperature growth conditions, the periodicity and interface abruptness of (InAs)_4_(GaAs)_3_/InAlAs MQWs grown using the MEE mode are better than those of InGaAs/InAlAs MQWs grown using the conventional MBE growth mode.

## 4. Conclusions

The use of the MEE mode has been demonstrated to be an effective method for the low-temperature growth of (InAs)_4_(GaAs)_3_/InAlAs MQWs on InP substrates, achieving high-quality quantum well structures with excellent optical properties. The migration-enhanced epitaxy technique facilitates growing (InAs)_4_(GaAs)_3_ MQWs at the substantially reduced temperature of 230 °C, avoiding mismatch dislocations. This demonstrates a promising approach to fabricate advanced optoelectronic devices with superior electron mobility and suppressed alloy scattering. The grown (InAs)_4_(GaAs)_3_/InAlAs MQWs exhibit strong photoluminescence responses and high room temperature electron mobility, indicating a good crystal quality and well-defined superlattice periods, which are crucial for applications in high-speed transistors, terahertz imaging, and optical communications.

The findings address the challenge of growing thicker InAs/GaAs superlattice layers for enhanced light absorption and provide insights into the material design for optoelectronic devices, particularly those requiring short carrier lifetimes such as terahertz photoconductive antennas and high-speed optical switches. This research contributes valuable knowledge to the field of low-temperature epitaxial growth techniques for III-V semiconductor materials and opens the door for further advancements in the development of high-speed optoelectronic devices.

## Data Availability

Data are contained within the article.
